# Selected Physicochemical Properties of Lyophilized Hydrogel with Liposomal Fraction of Calcium Dobesilate

**DOI:** 10.3390/ma11112143

**Published:** 2018-10-31

**Authors:** Ewa Pilch, Witold Musiał

**Affiliations:** Department of Physical Chemistry, Pharmaceutical Faculty, Wroclaw Medical University, Borowska 211, 50-556 Wroclaw, Poland; ewa.pilch@student.umed.wroc.pl

**Keywords:** liposomes, calcium dobesilate, lyophilization, dynamic light scattering, optical microscopy, Fourier-transform infrared spectroscopy

## Abstract

Lyophilization is the process of drying and improving the stability of various pharmaceutical preparations. In this work we evaluated the properties of 11 hydrophilic gels calcium dobesilate with liposomes based on soybean lecithin, subjected to the freeze-drying procedure. Liposomes were produced by using method thin lipid film. Lyophilization was carried out under conditions of temperature equal (−30 °C) and pressure 0.37 mbar. We evaluated the preparations with dynamic light scattering (DLS) method, optical microscopy and Fourier-transform infrared spectroscopy (FTIR). In this work we presented the average results for the particle diameter in the sample and PDI (polydispersity index) value for the samples that produced the results. When testing using the DLS method on a Malvern Zetaseizer, results for 7 samples were not obtained. Two of next four samples were characterized by an increased size of the liposome particle resulting from a lower concentration of ethanol compared to the rest of them. Three samples under the microscope did not show any differences. It was possible only to see single crystals probably of undissolved calcium dobesilate. Some clusters were observed in the 4 samples, and when they appeared they were very small. The aggregates and irregular liposomes present in the rest of the samples may have been formed due to the destabilizing activity of ethanol towards lipid membranes. In the FTIR spectrum for MC, the peak was observed at the wavenumber of ca. 2900 cm^−1^ and of about 1050 cm^−1^. In case of pure calcium dobesilate we observed low pick at the wavenumber of about 3400 cm^−1^. The spectrum has a low peak at the wavenumber of 1450 cm^−1^ and intense peaks ranging from approx. 1000 cm^−1^ to approx. 1200 cm^−1^. Decay of the lecithin peak in formulations with liposomes at 1725 cm^−1^ wavelength may indicate the occurrence of the hydrolysis reaction in the system. Probably there was a hydrolysis of the ester bond connecting the rest of the phosphoric acid and the choline with the glycerol residue.

## 1. Introduction

Transdermal and intradermal drug delivery is a convenient alternative to oral administration for drugs [1]. It reduces the frequency of adverse effects and increases effectiveness of recommended therapy by improving compliance of the patient [2]. However, the transdermal delivery of drugs has some limitations [3]. Currently, there are many methods to improve percutaneous drug delivery. To increase the permeability of the skin and thus increase the bioavailability of the active substance, methods associated with the vehicle and/or drug or associated with the modification of the epidermal layer are used.

The methods related to the preparation include encapsulation of the therapeutic molecules in liposomes. The methods associated with stratum corneum permeability include modification of skin lipid fluidity as well as application of chemical agents increasing skin permeation [4]. One of the agents increasing the penetration of the active substance by skin is ethanol. In the literature the most common is the addition of the 10–50% ethanol to obtain optimal effect [5]. Liposomes are small vesicles structurally similar to biological membranes. The drug molecules can be enclosed in the internal water space (this applies to hydrophilic compounds), or incorporated into the external lipid bilayer (this concerns lipophilic compounds) [6]. Liposomes may be used to solubilize poorly soluble drugs. Another premise for liposomes application is the formation of reservoirs for drugs that require a long time to release. Due to permeation of phospholipids through lipid layers of the cornified part of the epidermis, they act as drug penetration enhancers. In addition, controlled transdermal patches for releasing the drug substance can be developed using liposomes [7]. Favorably, liposomes are biodegradable and biocompatible particles and are characterized by low toxicity [8]. Liposomes may hydrated the surface of the skin according to the method of application and the structure of liposomes [9].

Hydrogels are used as carriers for medicinal substances due to their beneficial biological and physicochemical properties. The high water content in hydrogels favors biocompatibility and good tolerance [10]. Methylcellulose in aqueous conditions may represents thermoreversible semi-solid hydrogel, applicable for food, pharmaceutical, cosmetic, textile, and biomedical industries [11]. Liposomal hydrogels are intensively developed for controlled drug delivery via oral [12] and nasal mucous [13], as well as dermatological preparations [14].

Enhancement of formulation stability may be realized via freeze-drying, however the components, as well as the conditions may strongly influence the resulting product [15,16]. Lyophilization enables water removal in vacuum conditions, where the sublimation process takes place [17], especially in the case of thermolabile preparations [18]. Viruses, bacteria, vaccines, proteins, or colloidal carriers, i.e., liposomes, nanoparticles and nanoemulsions may be freeze-dried [19] using varied techniques of lyophilization [20]. The process influences solubility and bioavailability of Active Pharmaceutical Ingredients (API) [21] and the durability of pharmaceutical preparations [22,23]. The efficiency of the liposomes lyophilization process consists of product related parameters, such as a bilayer lipid composition, the size of liposomes, and the presence of lyoprotectants. Appropriate freezing and drying protocols and storage conditions of the obtained preparations also strongly influence the process [24]. Lyoprotectants glucose, fructose, trehalose [25], guar gum, or xanthan gum [26] stabilize the bioactive molecules in the course of the procedure. Various methods may increase the stability of liposomes, including freeze-drying, freezing, and spray drying [27]. Lyophilized liposomes are stable according to Gala et al. [28]. The size of each individual liposome as well as the ability to encapsulate API usually are preserved in these processes [29]. In comparative studies liposomes in the liquid form are the least stable, followed by lyophilizates without lyoprotectant. The most stable were lyophilizates containing sugar as lyoprotectant. These tests were carried out on samples stored at 4 °C and 25 °C [30]. The lyophilization process is usually carried at temperatures lower than (−20)–(−30) °C [17,31]. Researches from Ireland stabilized by lyophilization Lactobacillus rhamnosus GG (LGG). The bacterial strain was frozen at −22 °C or −43 °C for 21 h. Then for 5 h samples were stored at −80 °C. Lyophilization was carried out for time 48 h under pressure below 0.1 mbar and temperature below −40 °C. Lactose and trehalose were used as lyoprotectants [32]. Singh et al. carried out lyophilization at a temperature of −75 °C and pressure conditions of 10–13 mbar. Lyophilization improved the solubility of the resulting drug form compared to constant dispersion [33]. The preparation of lyophilizates can be used to increase the stability of the finished liquid form of the drug, what was evaluated in successful redispersion test [34]. Lyophilized liposomes are also obtained by modified reverse phase evaporation method (the so-called modified reverse-phase evaporation (REV) method), which minimizes decay of liposomes during lyophilization [35,36]. Lyophilization of hydrogels can be lead under similar conditions to the classical lyophilization of liposomes. Initially the sample is frozen at −20 °C or by immersing in liquid nitrogen (−196 °C) after gelation and dried below 10 mbar [37]. Lyophilized hydrogels retain their properties while improving the stability of the formulation during storage [38]. Marefati et al. investigated the effect of lyophilization on emulsions stabilized witch starch granules applying −50 °C, with pressure maintained at 0.01 mbar; the procedure resulted in well dispersible, oil-filled particles [39]. Research in Taiwan on enoxacin-containing liposomes was carried out with lyophilization lasting 24 h at −50 °C and 13 mbar [40]. Shah and Misra investigated lyophilized liposomes with amikacin. They lyophilized for 48 h with various lyoprotectants to achieve the effect of process optimization [41].

The conditions and precise parameters of lyophilization of liposomes, especially with regard to the time of the process and the presence of lyoprotectants is presented rather sparely, however the above mentioned sources confirm strong interest in this topic.

Calcium dobesilate (calcium 2,5-dihydroksybenzene sulphonate) has interesting pharmacological activity, including reduction of capillary hyperpermability, inhibition of platelet aggregation [42,43], and inhibition of prostaglandins and thromboxanes [44,45,46]. The safety of the drug was evaluated in details [47], and the skin application of a hydrophilic gel with liposomal fraction of calcium dobesilate may be an interesting option for dermal and transdermal delivery of the drug. Calcium dobesilate is easily soluble in water and ethanol [48], however the molecule is unstable in aqueous environment and may form ionic complexes with functional groups of drug carrier [49].

The aim of the work was to prepare hydrophilic gel of calcium dobesilate with liposomes based on soybean lecithin, subjected to the freeze-drying procedure and evaluation of selected properties of the product after rehydration.

## 2. Materials

Calcium dobesilate (Galena SPF, Wrocław, Poland), soy lecithin (ECOSPA, Warszawa, Poland), cholesterol (Wytwórnia Euceryny Laboratorium Farmaceutyczne “Coel”, Kraków, Poland), ethyl alcohol (Chempur, Piekary Śląskie, Poland) were used to prepare liposomes. The hydrophilic gels were prepared using methylcellulose of the viscosity of 4 Pas per 2% of aqueous solution at 20 °C (Sigma Aldrich, St. Louis, MO, USA). The entire technological process used deionized water from its own purification station (Wroclaw Medical University, Wrocław, Poland).

## 3. Methods

### 3.1. Preparation of Liposomes (in Reference to the Previous Publication)

Qualitative-quantitative compositions of the obtained preparations are presented in Table 1. 11 preparations containing the liposomal fraction with calcium dobesilate were developed in the work, and prepared in reference to the previous publication [50]. Shortly, the lipid substances were weighed and dissolved in an appropriate amount of chloroform. A thin lipid film was made by using a vacuum evaporator. The process was carried out over a 60 °C bath water and under pressure of 318 mbar at 5 RPM. Next the content of the flask were dried at 20 °C for 1.5 h in vacuum desiccator. After drying the preparation, the lipid film was hydrated with an aqueous or hydroethanolic solution with calcium dobesilate. The system was ultrasonically homogenized in 60 °C water bath, twice for 10 min.

### 3.2. Preparation of Lyophilizates

**Figure 1:**
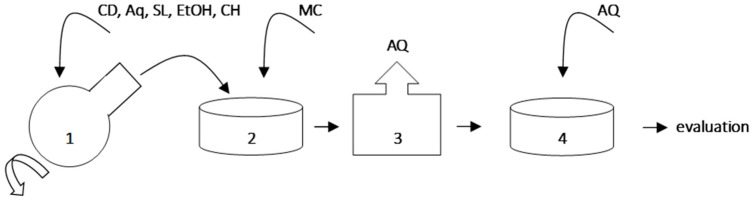
Overall procedure of samples preparation: CD—calcium dobesilate, MC—methylcellulose, EtOH—ethanol, SL—soy lecithin, CH—cholesterol, AQ—deionized water, 1–liposomes preparation, 2–preparation of methylcellulose gel with liposomal fraction, 3–freeze-drying, 4–rehydration of samples; the components were used according to Table 1.

Samples in liquid form were spilled with a thin layer on Petri dishes and protected from external factors by aluminum foil. Samples were frozen at temperature −80 °C. We did two lyophilization, each of them lasted 24 h. Between lyophilization samples were stored in a low-temperature freezer at −80 °C. Lyophilization was carried out under −30 °C and 0.37 mbar, using a Steris Lyophilizer Lyovac GT2. Preparations, which were obtained during the lyophilization, can return to the previous lipid form after addition of water without qualitative losses. The overall procedure of preparation of assessed samples A–K is presented on Figure 1.

### 3.3. DLS—Dynamic Light Scattering

The hydrodynamic diameter of the particles was examined via DLS (dynamic light scattering) what enabled, in limited field, evaluation of polydispersity of prepared samples of rehydrated dispersions of liposomal fraction according to procedures performed formerly in our department [51]. The measurement of hydrodynamic diameter of the particles was carried out using the Zetaseizer Nano ZS ZEN3600 analyzer from Malvern (UK). The particle analyzer was tested for the H, I, J, and K formulations prior to the lyophilization process. The test was carried out at 25 °C using a Polystyrene Latex material cuvette. Five measurements were taken and respective standard deviations were calculated.

### 3.4. Optical Microscopy

The samples were visualized by microscope photos using an optical microscope with camera and an Olympus BCX43 image analysis kit. The images were taken using the LCmicro program (Version 5.2, Olympus Soft Imaging Solutions GMBH, Münster, Germany).

### 3.5. FTIR—Fourier-Transform Infrared Spectroscopy

The study was performer on a Fourier-transform infrared spectrophotometer (FT-IR) and ATR model Nicolet iS50 (Thermo Fisher Scientific, Waltham, MA, USA). FTIR spectroscopy is used as a screening tool to determine the composition of a preparation having the desired properties [2]. Infrared spectroscopy can predict the behavior of penetration enhancers in relations to skin lipids [4]. A test was carried out for the standards, which were single auxiliary substances and calcium dobesilate and then for specific samples.

## 4. Results

### 4.1. DLS

Table 2 presents the average results for the particle diameter in the sample and PDI (polydispersity index) value along with the standard deviation for the samples that produced the results.

No measurement results were obtained for A–G samples. Sample H and sample J were characterized by higher standard deviation values than sample I and sample K. Sample H reached diameter in the range from 10,890 to 22,560 nm. The diameter of the liposomes of the sample I was in the range between 3324 nm and 9150 nm. The particle size of the J-sample liposomes was between 10,350 nm and 21,820 nm. Sample K reached a diameter between 4606 nm and 13,000 nm. The PDI value for tested sample H was in the range of 0.039–0.717. For sample I, the PDI value was 0.078–1. The J sample was characterized by a polydispersity index in the range of 0.498–1. The PDI value for liposomes from sample K was in the range 0.108–1.

**Figure 2:**
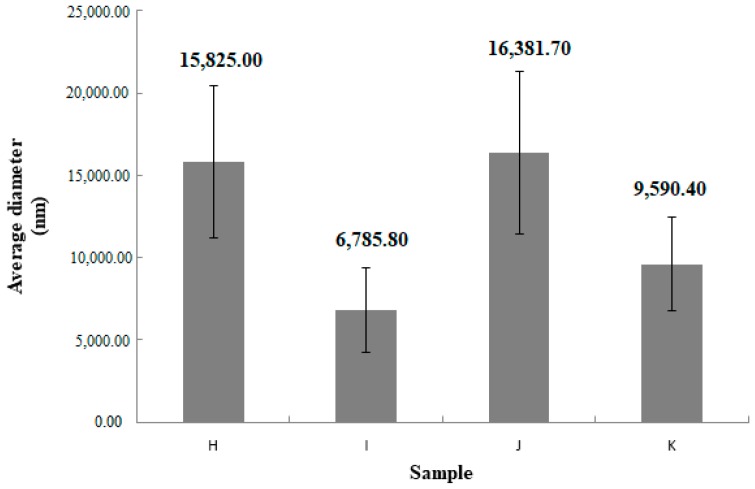
Obtained results for measurements of hydrodynamic diameter of liposome particles, n = 5, the Y-bars represent the standard deviation.

**Figure 3:**
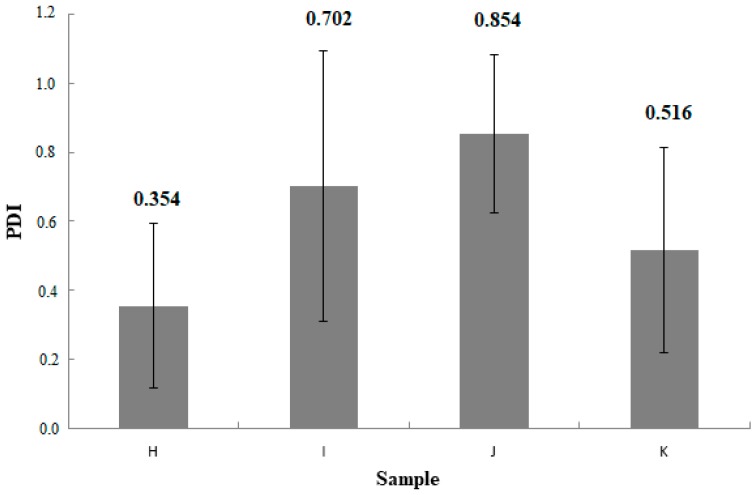
Obtained results for measurements of PDI of samples, n = 5, the Y-bars represent the standard deviation.

The average values of the measurements are presented on Figure 2 and Figure 3. The figures includes obtained deviation.

### 4.2. Optical Microscopy

**Figure 4:**
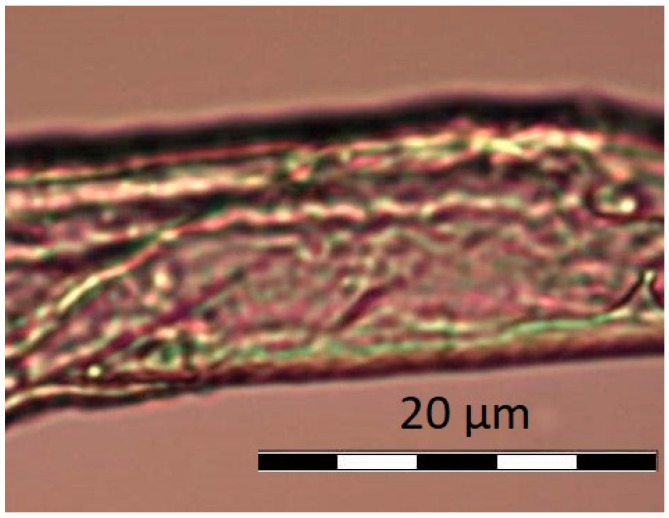
Undissolved crystal of calcium dobesilate.

**Figure 5:**
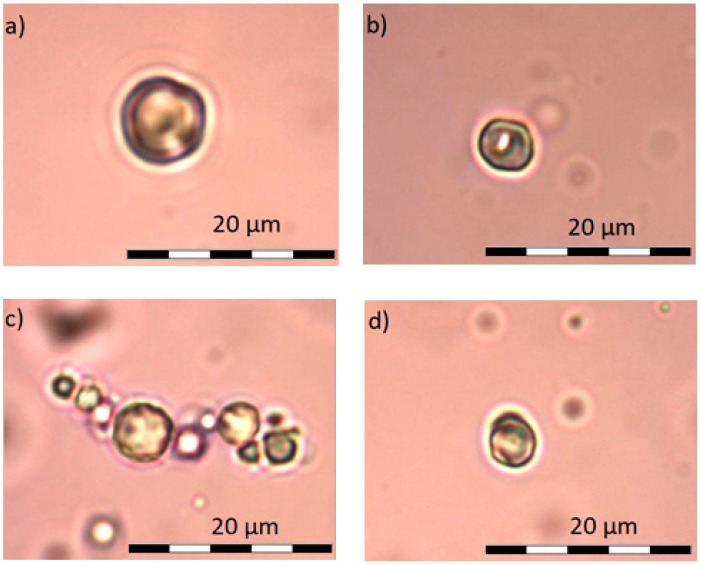
(**a**) liposomes D in zoom 400× (**b**) liposomes E in zoom 400× (**c**) liposomes F in zoom 400× (**d**) liposome G in zoom 400×.

**Figure 6:**
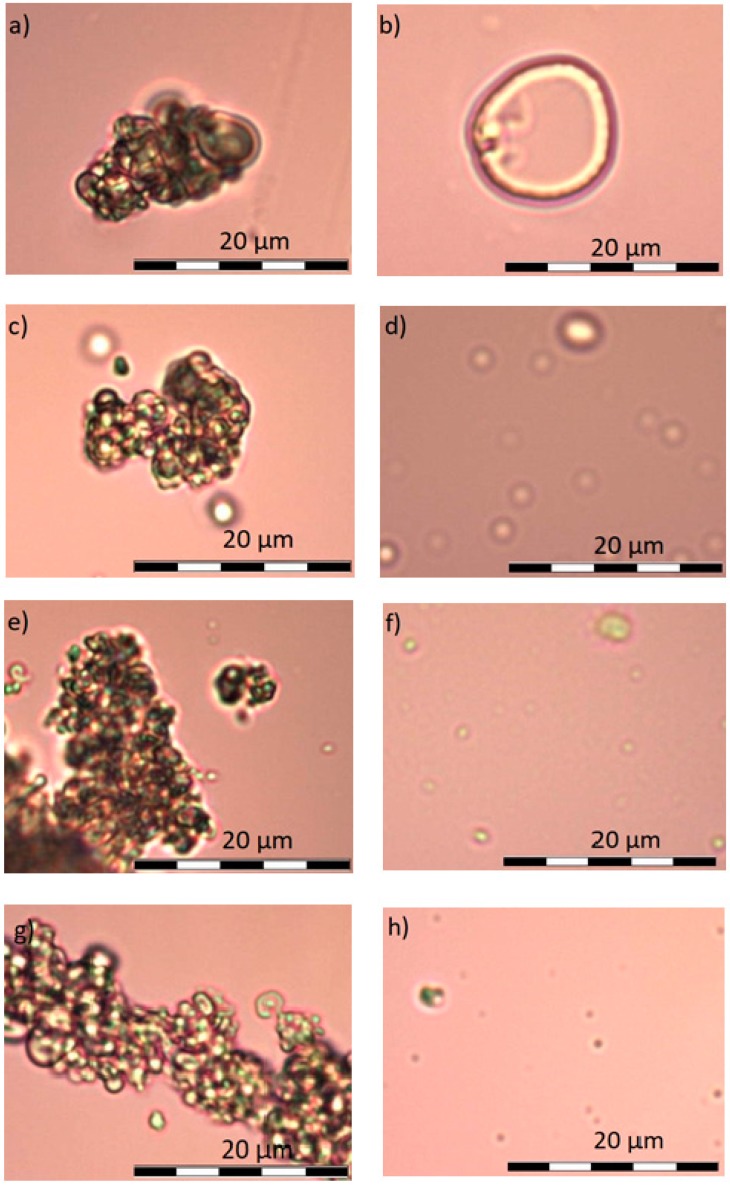
(**a**) liposomes H in zoom 200× (**b**) liposomes H after mixing and in zoom 400× (**c**) liposomes I in zoom 200× (**d**) liposomes I after mixing and in zoom 400× (**e**) liposomes J in zoom 200× (**f**) liposomes J after mixing and in zoom 400× (**g**) liposomes K in zoom 400× (**h**) liposomes K after mixing and in zoom 400×.

Obtained results were confirmed by microscopic photos using an optical microscope with camera and Olympus BCX43 image analysis kit. Samples A, B, and C under microscope do not show any differences. It is possible to see single crystals probably of undissolved calcium dobesilate, Figure 4. In the case of observation of the D–K, Figure 5 and Figure 6, liposome samples no crystals were observed. The pictures taken are shown in the figures below.

### 4.4. FTIR

**Figure 7:**
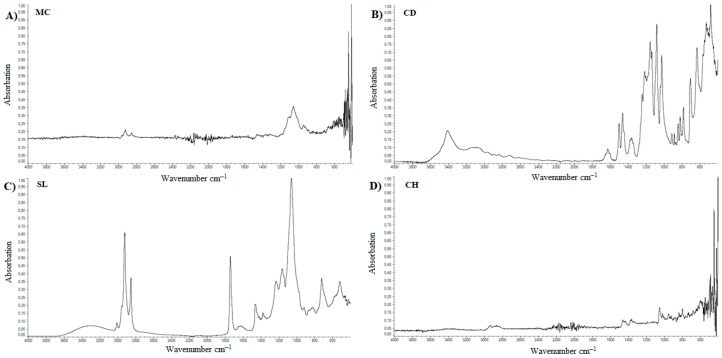
FTIR spectrum for standards. (**A**) methylcellulose; (**B**) calcium dobesilate; (**C**) soy lecithin; (**D**) cholesterol.

The standards and lyophilized samples were subjected to FTIR analysis. In spectrum for MC, presented on Figure 7A, the peak was observed at the wavenumber of about 2900 cm^−1^. In addition, there can be a characteristic peak at wavenumber of about 1050 cm^−1^. In case of pure calcium dobesilate, Figure 7B we observed low pick at the wavenumber of about 3400 cm^−1^. The spectrum has a low peak at the wavenumber of 1450 cm^−1^ and intense peaks ranging from approx. 1000 cm^−1^ to approx. 1200 cm^−1^. The basic components of liposomes are lipids. Soybean lecithin was used in this work. Spectrum FTIR for soy lecithin is presented on Figure 7C. A sharp and intense peak was observed at wavenumber 2900 cm^−1^. Sharp, short peak was observed at 1725 cm^−1^. A small peak is approximately 1625 cm^−1^. The most intense peak occurred at a wavelength of 1050 cm^−1^. In the wavenumber range 1100–1450 cm^−1^ we observed several smaller peaks. The FTIR spectrum for cholesterol—Figure 7D shows a peak for wavenumber of about 3000 cm^−1^. The region between 2800 cm^−1^ and 3200 cm^−1^ is characteristic of the presence of cholesterol in the sample. Penetration enhancer for API was ethanol. The ethanol testing was carried out. We did not present the results because during lyophilization it was mostly evaporated.

**Figure 8:**
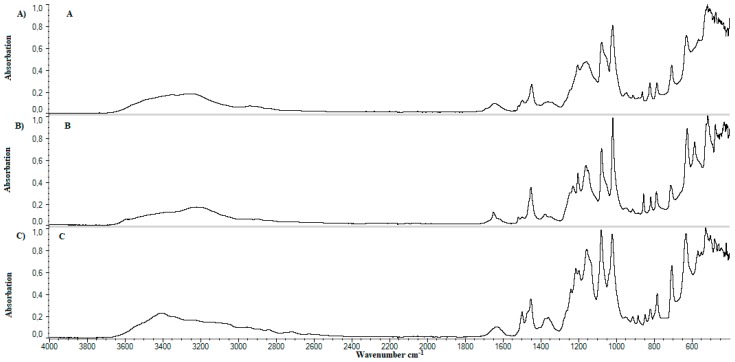
Comparison of spectrum FTIR for samples without cholesterol and lecithin. (**A**) Sample A; (**B**) sample B; (**C**) sample C.

**Figure 9:**
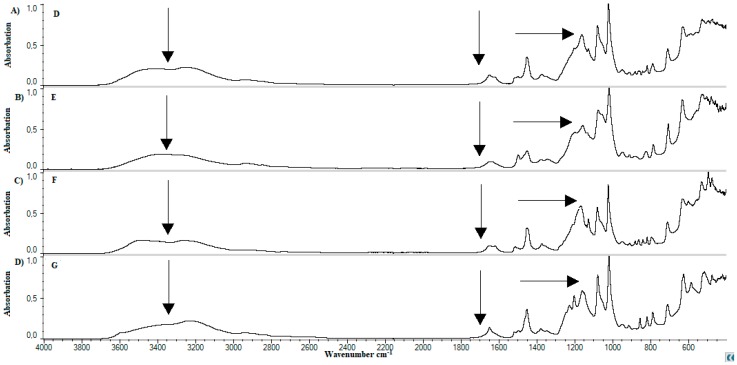
Comparison of Spectrum FTIR for samples D–G. (**A**) Sample D; (**B**) sample E; (**C**) sample F; (**D**) sample G.

**Figure 10:**
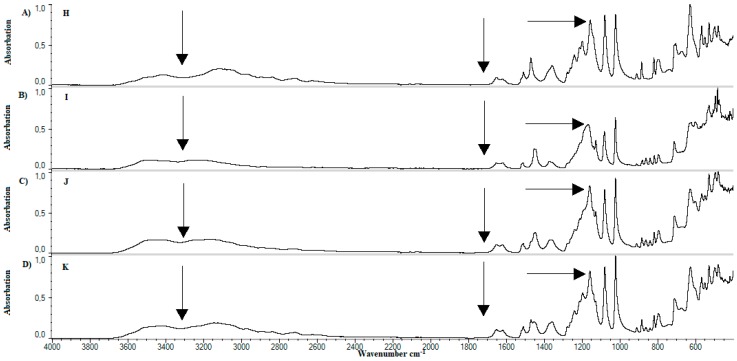
Comparison of Spectrum FTIR for samples H–K. (**A**) Sample H; (**B**) sample I; (**C**) sample J; (**D**) sample K.

After the analysis of standards, examination of prepared samples was carried out. Figure 8 shows the FTIR spectrum for samples A—Figure 8A, B—Figure 8B, and C—Figure 8C. A change in peak structure between wavenumbers 3000–3600 cm^−1^ can be noticed. A comparison of samples B, Figure 8B, D—Figure 9A, F—Figure 9C, H—Figure 10A, and J—presented on Figure 10C, contains 20 grams of ethanol and allows the observation that the peak in the range of the wavenumber 3000–3600 cm^−1^ is changed. Peak deriving from lecithin at the wavenumber of 1725 cm^−1^ disappears. Peaks occurring around wavenumber 1200 cm^−1^ differ in shape and sharpness. A comparison of spectra obtained allows the observation that in cases of samples with an increased content of ethanol, it is samples C—Figure 8C, E—Figure 9B, G—Figure 9D, I—Figure 10B, and K—presented on Figure 10D, a peak in wavenumber range 3000–3600 cm^−1^ is wider than standard. The shape of peak changes at 1200 cm^−1^. A peak deriving from lecithin at 1725 cm^−1^ disappears. The presence of methylcellulose in samples also affects on change in appearance of peaks. Visible change appears in the area of “finger print”.

## 5. Discussion

### 5.1. Hydrodynamic Diameter of Particles

When testing using DLS method on a Malvern Zetaseizer, results for samples A–G were not obtained. Concentration of 1.5% methylcellulose in samples A–G probably affected the disturbance of measurement results. According to the literature, the average size of methylcellulose particles in solution is about 20,000 nm [52]. In addition, the presence of filler reduces mobility of liposomes in medium of high viscosity. Those results were obtained only for samples not containing in their qualitative composition methylcellulose. Results are presented in Table 2 and on Figure 2 and Figure 3. Sample H and sample J were characterized by an increased size of liposome particles resulting from a lower concentration of ethanol compared to the other two formulations. The presence of a higher concentration of ethanol reduces the average particle size, which is confirmed in the literature [5,53]. Sample H and sample J are characterized by higher standard deviation values than sample I and sample K. Formulations J and K have a higher hydrodynamic diameter of particles with respect to formulations H and I. This is probably due to addition of cholesterol, which influences to increasing diameter of the particles, which is consistent with the literature data [54].

### 5.2. Optical Microscopy

On viewing the samples A–C, Figure 4, clusters were not observed. In some places, only undissolved crystals can be seen. They are probably calcium dobesilate crystals. Homogenity of the microscope image propably results from fact that none of the components shows incompatibilities among themselves. The present ethyl alcohol does not precipitate either methylcellulose or calcium dobesilate [48,55]. During the observation under the microscope, it was noticed that presence of gel results in lower tendency for aggregation by liposomes. Some clusters were observed in the D–G samples, presented on Figure 5, and when they appeared they were very small. When we were taking microscopic photographs, we found that liposomes take different shapes depending on the environment in which they are suspended. In an environment of high osmolarity, i.e., in a methylcellulose gel, they give off water and their surface becomes wrinkled and liposomes themselves show collapse characteristics. In an aqueous environment, i.e., in H–K formulations—Figure 6, due to the low osmolarity of surrounding environment, they maintain a spherical shape. The aggregates and irregular liposomes present in the preparations may be due to ethanol content performing destabilizing actions of lipid membranes [56]. After microscopic observation, it can be assumed that the relatively high PDI value in the measurements obtained with Zetaseizer results from the tendency of liposomes to aggregate.

### 5.3. FTIR

Lyophilized samples were subjected to FTIR analysis. In spectrum for MC, Figure 7A, tensile vibrations were observed from aliphatic C–H bonds with a wavenumber of about 2900 cm^−1^. In addition, tensile vibrations of C–O bonds and bending O–H bonds at a wavenumber of approximately 1050 cm^−1^ can be observed. Results are confirmed in the literature [57,58,59]. In the case of pure calcium dobesilate analysis—Figure 7B—we observe characteristic stretching vibrations of O–H groups at wavenumber of approximately 3400 cm^–1^. The spectrum has a peak at wavenumber 1450 cm^−1^, which indicates on the presence of stretching vibrations of S=O bonds. There are also tensile vibrations from C–O bonds at approximately 1100 cm^−1^ [60]. As predicted by FTIR spectra of soy lecithin, Figure 7C, a peak corresponding to the C–H stretching vibrations at 2900 cm^−1^ wavenumbers was observed. In addition, at 1725 cm^−1^ we observe stretching vibrations of those ester groups. A low intensity peak at 1450 cm^−1^ indicates on presence of bending vibrations of amide bond [61]. This peak also demonstrates the presence of the bond P–O [62]. Intense peak occurs at a wavelength of 1100 cm^−1^ and is responsible for occurring bending oscillations of those O–H groups and the stretching vibrations of the C–O bonds [61]. A faint peak of approximately wavenumber 1625 cm^−1^ probably derived from the vibrations of the stretch N–H bonds, is visible [63]. The FTIR spectrum for cholesterol—Figure 7D shows a peak for wavenumber of about 3000 cm^−1^. Region between 2800 cm^−1^ and 3200 cm^−1^ is characteristic of presence of cholesterol in sample. The presence of peak demonstrate occurrence of tensile vibrations of C-H bonds and vibrations from carbon cyclic rings [64]. A comparison of peaks described in the literature with those obtained is summarized in Table 3.

In the comparison of developed formulations, it was observed that the addition of ethanol affects flattening and widening of peak occurring in wavelength range of 3000–3600 cm^−1^. This is probably due to increased number of hydroxide bonds. Decay of lecithin (Figure 9 and Figure 10) peak at 1725 cm^−1^ wavelength may indicate occurrence of the hydrolysis reaction in the system. Probably there was a hydrolysis of the ester bond connecting rest of phosphoric acid and choline with glycerol residue. In effect existing liposomes can be maintained in their spherical form by long-chain fatty acids or their salts. Figure 9 and Figure 10 confirm the presence of calcium dobesilate in the system due to presence of a characteristic peak at wavelength 3400 cm^−1^.

## 6. Conclusions

The freeze-dried hydrogel with calcium dobesilate and liposomal fraction may be rehydrated, and the product contains liposomal particles. Swelled methylcelluse prevents the aggregation of liposomes, however they are deformed, probably due to high osmolarity of surrounding environment. The combination of ethanol and cholesterol influences the size and polidyspersity of prepared and rehydrated liposomes. As a result of rehydration of the preparation the lecithin-like peak disappears at the wave number 2800–3000 cm^−1^. It is not observed in ready samples, which may indicate the complexation and/or screening of these groups. In the case of combination of components, the hydrolysis of ester bonds in lecithin is likely to occur. This is due to the disappearance of the peak in the FTIR spectrum at the wavelength of 1725 cm^−1^. In the formulated preparations the homogenisation via sonification resulted in highly polydispersed fraction of liposomes.

## Figures and Tables

**Table 1:** Qualitative-quantitative compositions of the obtained preparations.

Formulation	Components (g)					
	CD	MC	EtOH	SL	CH	AQ
A	2.5	1.5	0.0	0.0	0.0	96.000
B	2.5	1.5	20.0	0.0	0.0	76.000
C	2.5	1.5	48.0	0.0	0.0	48.000
D	2.5	1.5	20.0	0.041	0.0	75.959
E	2.5	1.5	48.0	0.041	0.0	47.959
F	2.5	1.5	20.0	0.041	0.004	75.955
G	2.5	1.5	48.0	0.041	0.004	47.955
H	2.5	0.0	20.0	0.041	0.0	77.459
I	2.5	0.0	48.0	0.041	0.0	49.459
J	2.5	0.0	20.0	0.041	0.004	77.455
K	2.5	0.0	48.0	0.041	0.004	49.455

CD—calcium dobesilate, MC—methylcellulose, EtOH—ethanol, SL—soy lecithin, CH—cholesterol, AQ—deionized water.

**Table 2:** The average results for the particle diameter in the sample and polydispersity index (PDI) value along with the standard deviation (SD).

Sample	Average Diameter (nm)	SD	PDI	SD
H	15,825.0	4594.4	0.354	0.239
I	6785.8	2570.9	0.702	0.392
J	16,381.7	4958.2	0.854	0.230
K	9590.4	2854.5	0.516	0.299

**Table 3:** Peaks in FTIR for clean substances.

Lp.	Substance	References	Observed	Group	Literature
		cm^−1^			
1.	Methylcellulose	1126	1125	C–O (oxygen stretching)	[57]
		1647	-	C−O (carbonyl stretching)	[57]
		2843, 2932	2925	C–H	[57,58,59]
		3459	-	O–H	[57,58,59]
2.	Calcium dobesilate	822	800−925	Aromatic C–H	[60]
		1023, 1361	1050, 1100, 1350	S=O, C–O	[61]
		3155	3100	Aromatic C–H	[62]
		3431	3400	O–H	[63]
3.	Soy lecithin	970−1200	1050	P–O–C; PO_2_	[61]
		1145−1200	1150	PO_2_	[61,62]
		1540	1450	P–O–alkyl and amide groups	[62]
		1630–1640	1625	C–N, N–H	[63]
		1720–1765	1725	C=O	[61]
		2850–2960	2850, 2925	C–H	[61]
		3015	3015	=C–H	[61]
4.	Cholesterol	2800–3200	2850–2950	C–H	[64]
